# The search for a TNBC vaccine: the guardian vaccine

**DOI:** 10.1080/15384047.2025.2472432

**Published:** 2025-03-15

**Authors:** Cory Fines, Helen McCarthy, Niamh Buckley

**Affiliations:** School of Pharmacy, Queen’s University Belfast, Belfast, UK

**Keywords:** Triple negative breast cancer, immunotherapy, vaccine, p53, mRNA

## Abstract

Nearly 20 million people are diagnosed with cancer each year with breast cancer being the most common among women. Triple negative breast cancer (TNBC), defined by its no/low expression of ER and PR and lack of amplification of HER2, makes up 15–20% of all breast cancer cases. While patients overall have a higher response to chemotherapy, this subgroup is associated with the lowest survival rate indicating significant clinical and molecular heterogeneity demanding alternate treatment options. Therefore, new therapies have been explored, with a large focus on utilizing the immune system. A whole host of immunotherapies have been studied including immune checkpoint inhibitors, now standard of care for eligible patients, and possibly the most exciting and promising is that of a TNBC vaccine. While currently there are no approved TNBC vaccines, this review highlights many promising studies and points to an antigen, p53, which we believe is highly relevant for TNBC.

## Introduction

Triple negative breast cancer (TNBC) makes up to 20% of all breast cancer cases.^1^ Current treatment options are limited leading to poor overall survival rates. Currently, immunotherapy is a key target for the treatment of TNBC. There have been large advancements in the design of immunotherapies including the design of TNBC vaccines. This review will explore the current TNBC vaccines and propose what we think is the way forward in TNBC vaccine therapy.

## Triple negative breast cancer current state of affairs

Worldwide, breast cancer has become the most diagnosed cancer with ~15% of these cases being triple negative, which is defined by absent/low expression of the hormone receptors, estrogen receptor and progesterone receptor, and lack of amplification of human epidermal growth factor receptor 2 (HER2). Unlike hormone receptor-positive breast cancers,^1–3^ TNBC patients tend to be younger, premenopausal, and to be of black or Hispanic ethnicity.^4^ The majority of TNBC cases are basal-like, highly aggressive, and have a high recurrence rate.^5,6^ The lack of receptor expression has left chemotherapy, in conjunction with surgery and radiotherapy, the main form of treatment for TNBC for many years.^4^

Treatment of TNBC has proved to be challenging partially due to the heterogeneity of the disease, where 4–6 different subtypes have been identified^7–9^ with the basal-like subtypes being the most common.^10^ While TNBC patients have historically been treated with adjuvant chemotherapy, in recent years, neoadjuvant chemotherapy (NAC) has become more prominent.^11^ A variety of chemotherapies are used for the treatment of TNBC, commonly used in combination to increase efficacy. Anthracyclines such as doxorubicin or epirubicin have been a key player in the treatment of TNBC and is commonly used in combination with cyclophosphamide and taxanes.^12–15^ Other chemotherapies have shown efficacy in early-stage TNBC both as a monotherapy and in combination, including taxanes and platinum-based agents.^16,17^ While chemotherapy is the standard of care of TNBC, there is a heterogenous response which is best seen when using NAC where treatment response can directly be measured at resection and then linked to outcome. When given NAC, TNBC has a higher rate of pathological complete response (pCR) compared to non-TNBC patients.^18^ This higher initial response rate to chemotherapy appears to contradict with lower overall survival rates. This is known as the “TNBC Paradox” and may be explained by the fact that while pCR rates are higher, the survival rates of those with residual disease are significantly lower in TNBC cases compared to those with non-TNBC disease.^18^ TNBC has a lower overall survival in all stages of disease compared to non-TNBC patients and increases in disparity as the disease metastasizes.^19^ Metastatic breast cancer is uncurable and accounts for 90% of breast cancer related deaths.^20–23^ Over 40% of TNBC patients will develop distant metastasis^10^ and while ~26% of non-TNBC metastatic breast cancer patients will survive 5 years,^24^ once metastasis occurs in TNBC patients (mTNBC), patients only have a prognosis of 10–13 months.^25^

There have been new targeted drug developments for the treatment of TNBC. One target is the human trophoblastic cell surface antigen-2 (TROP2) which is overexpressed in a proportion of all breast cancer types, including over 80% of TNBC cases.^26,27^ These increased levels are correlated with a worse prognosis in breast cancer patients.^28,29^ In 2021, sacituzumab govitecan-hziy (Trodelvy), a TROP2 antibody-drug conjugant (ADC) was approved for use in TNBC. However, it is only approved for TNBC patients with unresectable locally advanced or mTNBC who have previously received 2 or more systemic treatments with one of them being for mTNBC.^30,31^ Another prominent therapy is poly-ADP ribose polymerase (PARP) inhibitors (PARPi) in the context of BRCA1/2 mutations. PARPs are important in both base excision single-stranded DNA repair, and when inhibited the cells accumulate single stranded breaks which leads to double stranded DNA breaks forcing the cell to rely on homologous recombination to repair these breaks.^32^ Approximately 5–10% of breast cancers are hereditary in which 25–28% are caused by a mutation in BRCA1/2.^33^ BRCA1/2 play a pivotal role in homologous recombination meaning patients carrying the mutated gene lack the ability to fix the increased double stranded breaks caused by PARPi, ultimately leading to cancer cell death.^34^ BRCA related breast cancers are found to be more aggressive than sporadic breast cancer cases and are typically higher grade and triple negative. Two PARPi have been approved for use in TNBC patients with BRCA1/2 mutations, olaparib and talazoparib.^34^ While these drugs have shown increased efficacy for these patients, these therapies are still only targeting a portion of patients, with only 10–30% of TNBC patients harboring a BRCA1/2 mutation.^35^

Despite the current advancements in combination therapies, these drugs target a limited population leaving a large population of TNBC patients in need of new therapies. Therefore, the tumor microenvironment as a whole must be examined. One major component is the tumor immune microenvironment, composed of a variety of immune cells including macrophages, monocytic derived suppressor cells, and T-cells.^36^ The use of immunotherapies targeting these cells have become a focus of research for cancer in recent years with multiple approvals for a range of cancers including bladder, lung, melanoma, and lymphoma.^37^ Breast cancer immunotherapy was slower to gain traction as they were traditionally seen as “immune cold” tumors;^38^ however, TNBC has been found the most immunogenic breast cancer subtype,^39^ making it a good candidate for immunotherapy treatment.

## Current immunotherapies

One of the most studied immunotherapy targets is the programmed cell death receptor 1 (PD-1) and its ligand, programmed cell death ligand 1 (PD-L1). Normally, PD-1 is found on T-cells, NK cells, B cells, monocytes, and dendritic cells and binds to PD-L1 found on antigen presenting cells (APCs). When an immune response is no longer needed, PD-1/PD-L1 binding suppresses the immune system, serving as an immune checkpoint and maintaining immune homeostasis.^40^ In cancer, these cells, as well as cancer cells themselves, are known to increase expression of PD-1/PD-L1 inhibiting T-cell receptor (TCR) dependent activation of T-cells, thus helping to escape the anti-tumourigenic immune response.^41^

Antibodies capable of targeting PD-1 or PD-L1, known as immune checkpoint inhibitor (ICIs), help avoid suppression of T-cell function, thus enhancing tumor killing. Patients are tested for PD-L1 positivity before treatment which is defined as a tumor proportion score >1% or a combined positive score of >10.^42^ Atezolizumab is an anti-PD-L1-antibody that was approved by the FDA through accelerated approval for treating unresectable locally advanced and metastatic TNBC, which showed an increase in OS when administered in combination with chemotherapy in IMpassion130 clinical trial.^43^ The continued approval was based on the forthcoming results from the following study Impassion131, which had the same study design but replaced Abraxane for Taxol. This study showed no difference in PFS or OS when using Atezolizumab in combination with chemotherapy, and approval was withdrawn in August 2021.^44^ On the other hand, in July 2021 the FDA approved pembrolizumab (Keytruda), an anti-PD-1-antibody, in combination with neoadjuvant chemotherapy for the treatment of high-risk early-stage TNBC.^45^ In combination with chemotherapy, pembrolizumab has shown to increase efficacy over neoadjuvant chemotherapy alone and has become the standard of care for TNBC for patients with PD-L1 positive tumors.^46,47^

While ICI therapy usage has become widespread, they are limited by the heterogeneity of TNBC tumors and PD-1/PD-L1 expression in them. Lymphocytic positive breast cancer (LPBC), defined as tumors with > 50% lymphocytic infiltration and is typically found in TNBC patients, is associated with increased survival.^48,49^ High TIL scores have been associated with an increased response to ICI in TNBC patients.^50^ Despite this, LPBC positive patients only make up a small portion of TNBC patients (~20%).^51^ Furthermore, only 20–60% of TNBC tumors are PD-L1 positive.^52–56^ Moreover, the patients that are PD-L1+ are already the ones that typically have a greater response to chemotherapy.^57^ Patients can also have primary or acquired resistance to ICI, again limiting the effectiveness of these drugs.^58,59^

Therefore, while PD-1/PD-L1 antibodies have shown success in TNBC patients, there is still a need for further advancements. There are currently many ongoing immunotherapy clinical trials for the treatment of TNBC covered in various reviews;^60,61^ however, none that we know of have been approved for clinical use. We believe cancer vaccines are the future for TNBC treatment. Cancer vaccines date back to 1980 and have the potential to not only target a broad population but also have the ability to be personalized to individual patients.^62^ In the next section, we will discuss the design and possible precursors to cancer vaccine success and the current landscape of TNBC vaccines.

### Vaccines

There are a variety of different modalities of cancer vaccines including cellular-based (whole tumor cells, dendritic cells, etc), protein-based (whole protein, peptides, etc), nucleic acid based (DNA, mRNA), and vector-based (bacterial, viral, etc).^63,64^ While many cancer vaccines are in clinical trials, to date only 4 preventive and 2 therapeutic cancer vaccines are clinically approved.^65^ Out of the 4 preventative vaccines, 3 are for Human Papillomavirus (HPV) (Cervarix, Gardasil and Gardasil-9) and prevent cancer indirectly by preventing the infection of HPV that can cause cervical, head and neck, penile, vulvar, and vaginal cancer development.^66^ The other preventative vaccine also acts indirectly and is against Hepatitis B virus (HBV) and HBV related liver cancer.^67^ Bacillus Calmette-Guérin (BCG), a whole-cell tuberculosis preventative vaccine,^68^ is also used as a therapeutic vaccine in a large proportion of bladder cancer patients.^69^ BCG uses nonpathogenic *Mycobacterium bovis* bacteria, which is injected into the bladder, to stimulate the immune system in a non-antigen specific manner.^70^ The second therapeutic vaccine is Sipuleucel-T (Provenge), a vaccine for metastatic castration-resistant prostate cancer patients, composed of patients’ own dendritic cells stimulated with GM-CSF and the prostatic acid phosphatase antigen.^71^

### Preventative and therapeutic vaccines

Preventative and therapeutic vaccines function by primarily activating the humoral immune response or the innate immune response respectively. A preventative vaccine presents an antigen to activate a humoral (antibody-mediated) response involving the activation of B-cells. This can be attained without cell-mediated immunity; however, to have a robust and lasting immune response, B-cells need to be activated by T helper (CD4) cell. CD4 cells have two subsets, Th1 and Th2, which stimulate cell-mediated and antibody-mediated responses, respectively. The Th2-mediated response will stimulate B-cells into plasma and memory cells. Plasma cells will create antibodies including the first responders IgM, and the more specific and neutralizing antibody IgG. Memory cells will remain in the lymph nodes to respond to an immune response when the antigen is seen again. This response will be more rapid and mostly consists of specific IgG antibodies. In contrast, for a therapeutic vaccine, the focus is solely on the cell-mediated response. Successful therapeutic vaccines will introduce an antigen in which antigen presenting cells (APC), such as dendritic cells and macrophages, can digest and present using MHCII and MHCI molecules for the activation of CD4, majority Th1, and CD8 cells respectively.^72,73^ Human leukocyte antigen (HLA) regions HLA-A, B, and C encode for MHCI while HLA-DR, DQ, and DP encode for MHCII. A large amount of peptide vaccines tend to be HLA class dependent to increase efficacy; however, due to people expressing different HLA regions, this limits the patient population that benefit from these treatments. The presence of HLA-A and B/C is required for a successful therapeutic vaccine. HLA-A2 restricted vaccines are the most popular due to HLA-A2 being the most prevalent MHCI allele family in humans (~50% of Caucasians and ~ 35% of African-Americans).^74^

The most investigated antigen in BC is the human epidermal growth factor receptor 2 (HER2),^75^ expressed at low levels in normal cells and is overexpressed in ~ 20% of breast cancer cases.^76^ Two HER2 derived vaccines have, to date, progressed to clinical trials for the prevention of breast cancer recurrence; GP-2 (NCT03014076, NCT00524277) and AE37 (NCT00524277).^77^ GP-2 (NCT03014076), a HER2 peptide vaccine combined with GM-CSF treatment, underwent a Phase IB clinical trial in HLA-A2+ or A3+ HER2+ breast cancer patients^78^ and progressed to a Phase II trial which suggested patients that overexpress HER2 could benefit from a full vaccination treatment.^79^ There is currently a phase III trial investigating GP2 in combination with GM-CSF in HER2+ breast cancer patients (NCT05232916) expected to be completed in 2026. AE37, an HLA unrestricted modified HER2 peptide vaccine was tested in HLA-A2+ breast cancer patients in combination with GM-CSF in a phase II clinical trial. While there was no overall benefit seen in the vaccinated group, there seems to be a benefit in patients with low HER2 expression and TNBC.^80,81^ Another HER2-derived peptide vaccine, E75 also known as nelipepimut-S (NP-S) or even better known as NeuVax, has been evaluated in a Phase I-III clinical trials (NCT00841399/NCT00584789/NCT01479244).^77^ HLA-A2+ or A3+ and low HER2 expression breast cancer patients were treated with E75 in combination with GM-CSF monthly for 6 months and then every 6 months until 36 months. No significant difference was seen in disease free survival (DFS) between the vaccinated and placebo groups.^82^

### Antigen selection

Cancer antigens are typically found in two groups: tumor-associated antigens (TAAs) or tumor-specific antigens (TSAs). TAAs arise from proteins that are normally expressed in healthy cells but, in cancer, are genetically amplified, expressed in other organs, or have a post-translational modification affecting their protein levels.^83^ Since these antigens can be found in normal cells in lower levels, they have a lower affinity for T cell receptors (TCR), have undergone immune tolerance, and therefore have lower immunogenicity and a less effective antitumour response compared to TSAs which are not found in normal cells.^84^ Most TSAs arise from mutations that create proteins that are not normally found in cells, known as neoantigens. Neoantigens are completely foreign to the immune system making them less likely to be inhibited by immune tolerance, making them the ideal target for immune therapy.

### Impact of genetic factors on vaccine success

Tumor mutation burden (TMB), the total number of somatic mutations in a region of tumor genome, has been linked to the immunogenicity of (breast) cancer.^85^ It is thought that highly mutated tumors are more likely to have neoantigens and thus are targets of immune response. The p53 protein has been shown to be the most highly mutated protein in breast cancer tumors with a frequency ranging from 15 to 74%.^86,87^ It also has been shown that TNBC tumors have a higher frequency (up to 80%) of p53 mutations.^88–90^ In a study of 465 Chinese TNBC patients, 25,713 different somatic mutations were found with the most prominent mutations being TP53 (found in 74% of tumors).^91^

Furthermore, a higher median TMB is more common in TNBC in comparison to HR+ breast cancer.^50,92^ Mutations in DNA checkpoint/repair and cell replication pathways have been shown to increase the somatic mutation rate in the tumor and be associated with higher TMB and higher TIL.^93^ BRCA1 is a key DNA repair gene functioning primarily in homologous DNA repair. Eighty percent of breast cancer patients with a BRCA1 mutation are diagnosed as triple negative,^94^ making up to 10–30% of all TNBC cases.^35^ In comparison with wild type patients, those with BRCA1 mutations have been shown to have higher tumor specific neoantigens which stimulate the recruitment of T cells into the TME.^95^ Furthermore, 60–69% of TNBC patients who do not have a BRCA mutation, still have a similar phenotype, known as “BRCAness”.^41^ Patients with either BRCA1/2 mutations or “BRCAness” are more immunogenic than TNBC tumors without these genetic mutations and phenotype.^41^

While some studies have shown no clear association between TMB score and survival after immunotherapy treatment,^96^ others have shown a high TMB score in combination with a favorable immune subclass, one with higher CD8+ T-cell infiltration, was associated with a better survival in breast cancer patients.^97–99^ Altogether, TMB should be used as a supplementary predictor in combination with other immune type classifiers, such as PD1/PD-L1 expression.^100^ Therefore, TNBC can be seen as a good candidate for not only immunotherapy, but also for vaccine therapy.

### Cancer vaccine limitations and adverse events

While the focus of this review is to highlight the advancements of TNBC vaccines, it is also necessary to highlight some key general limitations of cancer vaccines although a more in-depth review of this is available.^101^ A major limitation of the effectiveness of cancer vaccines is the variability of response due to the heterogeneity of genetic factors aforementioned to in the previous section. Furthermore, there are various mechanisms of “immune escape/evasion”, which in turns limits the efficacy of vaccines. For example, tumors are able to increase immunosuppressive cells such as Tregs, MDSCS and “M2-like” macrophage, change cytokine expression and increase immunosuppressive properties (e.g. PD-L1 expression) leading to a Th2 dominant environment all while increasing angiogenesis and tumor growth.^102^ Moreover, cancer cells can lose MHC and antigen presenting machinery as well as decrease the targeted antigen expression.^103,104^

As highlighted in the coming sections, it is desirable to choose an antigen that is applicable to a wide patient population. Many off the shelf vaccines, whether TAAs, TSAs or viral antigens, are limited to a small patient population while personalized vaccines are neither time nor cost effective.^105^ Therefore, there is an unmet need for the development of shared tumor antigens that will broaden the population that will gain benefit from the vaccine. Lastly, there are possible adverse reactions when giving cancer vaccines. For instance, the delivery method (peptide,^106^ liposome,^107^ adenovirus,^108^ etc.) instead of to the antigen itself can induce an immune response or cause toxicity. Possibly worse, when using TAAs there is the potential of triggering an immune response against self-antigens found in healthy non-cancerous cells.^84^ Off-target immunogenicity and toxicity can be limited by utilizing neo-antigens/TSAs^109^ and modifying the carrier.^64^ While there are limitations and possible adverse reactions and toxicities to cancer vaccines,^101^ there has been much progress, including for the treatment of TNBC, which will be explored in the coming sections.

### TNBC therapeutic vaccines

Breast cancer is one of the most studied tumors for vaccine therapy.^110,111^ As previously discussed, HER2 is the most investigated protein for breast cancer patients; however, TNBC patients lack the overexpression of HER2 thus additional antigens need to be investigated with many pre-clinical trials with various antigens and delivery forms.

#### Pre-clinical

One highly studied antigen *in vivo* is Mucin 1 (MUC1), an epithelial glycoprotein highly expressed in over 90% of breast cancer patients.^112–114^ One group conjugated MUC1 to a carrier protein CRM197, a nontoxic mutant of diphtheria toxin, as a vaccine in a 4T1-Luc TNBC model.^115^ This study showed that weekly immunization slightly decreased the tumor size, measured by Luciferase bioluminescence, while significantly reducing T regulatory cells and Macrophage/M2 populations and increasing CD4+ T-cell populations.^115^ Another group designed Lipid/calcium/phosphate (LCP) nanoparticles encapsulating MUC1 mRNA in combination with an anti-CTLA-4 monoclonal antibody.^116^ In a 4T1 tumor model, both treatments alone elicited an increase in CD8+ cells measured by flow cytometry which increased when in combination leading to a significant decrease in tumor volume.^116^

Dendritic cells (DC) are the most prominent APC and can be collected from patient blood and stimulated to recognize certain antigens and re-delivered to patients. As previously discussed, Sipuleucel-T (Provenge) is a vaccine that is a clinically approved for prostate cancer. Sipuleucel-T is comprised of prostatic acid phosphatase and GM-CSF recombinant protein and is incubated with patients’ APCs ex vivo and then reinjected back into the patients.^117^ Using a similar approach, the Runt-associated transcription factor 2 (RUNX2) has been studied for TNBC. RUNX2 is an oncogene that has increased expression in BC and has been associated with cancer progression through various mechanism including inhibiting p53 mediated apoptosis. RUNX2 transfected DC were able to induce a Th1 response and create circulating T cells (CTLs) able to increase cytotoxicity in MDA-MB-231 TNBC cells compared to control DC in an *in vitro* setting.^118^

Other groups have focused their attention on creating nucleotide-based (DNA and mRNA) vaccines. Li et al. sequenced 4T1.2 and E0771 tumors and designed DNA vaccines encoding a mutant form of ubiquitin (Ub^mut^) specific for each tumor (Ub^mut^-4T1.2 DNA and Ub^mut^-E0771 DNA).^119^ In an E0771 tumor model, anti-PD-L1 decreased tumor volume while the Ub^mut^-E0771 DNA vaccine did not significantly reduce tumor volume alone. However, there was an increased anti-tumor effect when the vaccine was given in combination with anti-PD-L1 therapy. Unlike E0771, 4T1.2 was shown to not respond to anti-PD-L1 monotherapy. Meanwhile, the vaccine alone did decrease tumor volume, however not to a statistically significant level.^119^

#### Clinical

STEMVAC is a plasmid DNA vaccine which targets endoglin (CD105), YB1, SOX2, Cadherin 3 and MDM2. A phase I dose escalation study (NCT02157051) was performed in HER2- BC patients (25% being TNBC), where a Type I T-cell response was measured.^120^ A phase II study investigating STEMVAC in early-stage TNBC is currently recruiting patients (NCT05455658).

The “Mutanome Engineered RNA Immuno-Therapy (MERIT)” consortium is a collaboration of five European partners (academia and industry) seeking out a personalized approach for TNBC treatment including a mRNA vaccine.^121^ MERIT created a program to develop, validate, and manufacture drugs for individual patients (MUTANOME) as well as creating a storage bank of mRNA vaccines of the most frequently shared TAA in TNBC (WAREHOUSE).^122^ This concept moved to a phase I clinical trial (NCT02316457) testing WAREHOUSE vs MUTANOME or a combination in TNBC patients who had received surgery and neoadjuvant chemotherapy. Preliminary results of this study demonstrated that the mRNA vaccine pool led to an effective T-cell immune response to individual neoepitopes.^123^ The study is now complete, but the results have not yet been published.

Various peptide vaccines have also been explored for TNBC. PVX-410 is tetra-peptide vaccine used in HLA-A2+ TNBC patients that targets XBP1 (2 forms), CD138 and CS-1. PVX-410 in combination with other drugs has been tested in phase I clinical trials in both stage II/III (NCT02826434) and metastatic HLA-A2+ TNBC patients (NCT03362060). PVX-410 had low toxicity and induced an antigen specific response in both early TNBC^124^ and mTNBC^125^ patients allowing the study to move to an ongoing phase II (NCT04634747). TriAdeno is another multi-peptide vaccine that targets four different tumor antigens: CEA, Brachyury, six RAS mutations and MUC1. TriAdeno underwent a Phase I clinical trial in advanced cancer (NCT03384316) that showed tolerability and generation of antigen specific T-cell responses.^126^ Another Phase I study was carried out in second-line or greater metastatic TNBC patients (NCT03387085), and while preliminary results showed promising survival responses to the therapy,^127^ the study was terminated and the patients are no longer receiving intervention.

Tumor-associated carbohydrate antigens (TACAs) are broad-spectrum targets, and immunization with carbohydrate mimetic peptides (CMPs) can induce TACA-reactive antibodies to inhibit tumor cell survival.^128^ One CMP that has made it to clinical trials is P10s which was conjugated to the Pan T-cell carrier PADRE (P10s-PADRE).^128^ This was given with Montanide ISA 51 VG, a CTL enhancing agent,^129^ in the adjuvant setting in stage IV BC patients^130^ (NCT01390064) as well as in the neoadjuvant setting in ER+ patients (NCT02229084) and TNBC patients (NCT02938442). These phase I studies showed that P10s-PADRE is safe and elicits an immune response.^130,131^ It was found that the best schedule in the neoadjuvant setting is 3 doses given a week apart and completed 1 week before chemotherapy treatment.^131^ Another peptide vaccine explored for the used in TNBC is TPIV200, a Folate Receptor Alpha (FRα) peptide vaccine combined with GM-CSF. TPIV200 has shown to induce FRα specific T-cell responses in ovarian cancer patients.^132^ In a Phase II clinical trial (NCT02593227) completed in 2021, TPIV200 was given to stage I-III TNBC patients. While results have yet to be posted, a further Phase II double-blind study has been started (NCT03012100). Two other peptide vaccines for the treatment of TNBC that have been designed and yet to be furthered studied targeting Sema4A^133^ and SOX9.^134^

### TNBC preventative vaccines

#### Pre-Clinical

One target that has been studied for the development of a prophylactic/preventative vaccine is topoisomerase 2 alpha (TOP2A), an important enzyme in DNA replication, cancer cell proliferation and is a direct or indirect target of anticancer agents.^135^ Using the Cancer Genome Atlas, Lee *et al*. found that TOP2A was highly expressed in TNBC patients.^135^ Various TOP2A peptides were tested for Th1 induction efficiency and 3 were selected for the formation of a multi-peptide vaccine. Mice were treated with a TOP2A multi-peptide vaccine 6 times over 18 days before inoculation with TOP2A overexpressing mammary malignant cells (M6).^135^ Mice that were immunized had an average tumor volume of 11 mm^3^ 49 days after inoculation while the control group had an average volume of 736 mm^3^, a 67× fold change.^135^ Similarly, mice were immunized with a TPX2 microtubule nucleation factor derived multi-peptide vaccine 4 times before 4T1 tumor transplantation.^136^ Vaccinated mice had a lower tumor volume, reducing the average volume from 804 to 505 mm^3.136^ Similarly, a peptide vaccine against Survivin, an apoptosis inhibitor, was used to vaccinate mice before 4T1 tumor establishment, which led to a reduction in tumor growth.^137^ There is a report of a Survivin clinical trial, despite showing promising results there is limited details published.^138,139^

#### Clinical

One exciting development in TNBC vaccines is the use of α-Lactalbumin as an antigen, which is found on TNBC cells but not normal non-lactating breast cells.^140,141^ In 2010, Tuohy *et al*. published their findings on the α-Lactalbumin vaccine in murine breast cancer;^140^ however, there was concern about the vaccine’s translatability into humans. They addressed these concerns in a follow up paper in 2016,^141^ and now it is currently in a Phase I clinical trial (NCT04674306) both as a preventive and therapeutic vaccine. Thus far, 45 people have been enrolled and the maximal tolerated dose has started to be determined^142^ with an expected end date of later this year (2024).

### Current state of TNBC vaccines

Great progress has been made toward a TNBC vaccine with an encouraging push for patient specific vaccines with the work from studies such as MERIT; however, the cost and time associated with this and how that impacts patient care are a large concern. Furthermore, currently most of the antigens that are being explored are still for a very limited population within TNBC. In the next section, an antigen that not only encompasses a larger set of TNBC patients but also has potential to be used in various cancer types is proposed.

## The guardian vaccine

An antigen that has been highly investigated and shown promise is TP53 (p53). p53 is an important regulator of cell cycle, DNA repair, cell cycle regulation, apoptosis and senescence and has been deemed the “guardian of the genome”.^88^ As previously mentioned, the p53 protein is mutated in over half of human cancers (mutp53) and more specifically in ~ 30% of breast cancers^88^ and in up to 80% of TNBCs^143,144^

Over 75% of p53 mutations are missense mutations,^145^ those that cause a change in single amino acids, and typically occur at exons 4–9 making up ~190 codons, which encode the DNA binding domain. While non-missense mutations will end up producing no p53 protein, missense mutations form a mutant protein that has loss of function (LOF) of p53’s transcription factor and tumour suppressive activity which occurs in ~ 80% of p53 mutations in breast cancer.^146^ These LOF mutations drive cell proliferation and survival and thus metastasis^147^ and have been correlated with chemoresistance and lower efficacy of anti-tumour agents.^148,149^ Out of the 190 codons, 10 locations make up 30% of all missense mutations and are known as the “hotspot” mutations. These “hotspot” mutations are known to not only have LOF but also give mutp53 gain-of-function (GOF) activity based on how they interact with other proteins.^150^ There are 2 main forms of GOF missense mutations: contact mutations and structural mutations. Contact mutations disrupt p53’s ability to bind DNA which include residues R248Q, R237H and R273C. Meanwhile, structural mutations destabilize the p53 structure and reduce its thermostability which can lead to misfolding and again lack of DNA binding, this includes residues R175H, G245S, Y220C and R249S.^151^ Due to these types of mutations, p53 can now bind to and inhibit tumor suppressors (e.g. p63, p73)^152^ and enhance survival and proliferation.^151^ This newfound GOF activity can increase genomic instability, resistance to pro-apoptotic signals and drug resistance, tumor aggressiveness, metastatic potential and can promote TGF-β pro-tumourigenic properties.^153^

For mutp53 to have GOF activity, there are two key steps. Firstly, the mutant p53 allele must assert dominant-negative (DN) functions, antagonizing the remaining WT p53 allele leading to a loss of heterozygosity (LOH).^154^ It has been shown that without LOH, there is no protein stabilization and no GOF.^155^ Which leads us into the second step, protein stabilization which is necessary for p53 GOF activity. Due to its rapid ubiquitination by its main E3 ligase MDM2 and subsequent proteasome degradation, WT p53 is normally kept at very low levels. However, mutp53 is less susceptible to degradation leading to increased protein levels.^153^ The mechanism of how mutp53 is stabilized is still not fully known, however the heat shock protein complex HSP40/HSP70/HSP90 plays a major role in the stabilization of mutp53.^156^ HSP40, a co-chaperone of HSP70, is a first line defense detecting misfolded proteins and its various classes play various roles in stabilizing mutp53 protein in cancer. Specifically, DNAJA1/HDJ2 binds to misfolded mutp53 and prevents its CHIP ubiquitin ligase-mediated proteasomal degradation. On the other hand, DNAJB1/HDJ1 binds and stabilizes MDM2, the forementioned ubiquitin ligase of p53. This binding interaction inhibits MDM2 function on p53 and thus increased p53 protein levels.^157^ HSP90 also plays a role in stabilization of p53 by binding and inhibiting CHIP and MDM2.^158,159^ While the increased levels of mutp53 allows for GOF activity, it also unlocks a new target for immunotherapy.

During embryo development, proteins in the thymus are digested into peptides and presented via MHCI to T-cell Receptors (TCRs). This process is known as “self-tolerance” and is important for the body to learn how to discriminate between non-threatening self and foreign non-self-antigens.^160^ While some p53 peptides are presented and mediate negative selection of CD8+ T-cells, other peptides are either not presented or have low interaction with a TCR allowing for positive selection, thus becoming self (auto) antigens also known as cryptic determinants.^161^ However, these p53 self-antigen/peptides are not normally detected by the immune system due to being kept at low levels and normal expression being restricted to the nucleus.^162^ On the other hand, due to the increased levels found in tumors, p53 now has increased possibility of being presented. Either the cryptic determinants can be presented or the new mutp53 regions (found in the DBD).^162^ Multiple p53 determinants have been found from the “hotspot” regions of the DBD^163^ with mutant specific T-cell receptors, with p53 R175H being the most studied.^164–166^

This unmasking of p53 determinants is confirmed both by the activation of the humoral and cell-mediates innate immune response. A humoral response was confirmed by the detection of p53 antibodies in the serum of breast cancer patients^167^ while none were seen in healthy women. However, p53 follows the immunodominant rules of other self-antigens, whereby the immune response, creation of antibodies, is only elicited against a few peptides of the many formed. The N-term and C-term have been found to be the immunodominant determinants.^168^ Schlichtholz and team used both immunoblot and peptide mapping to show that despite the “hotspot” neoantigens of the DBD, p53 antibodies found in the serum were predominantly targeting the immunodominant epitopes, the amino and carboxy termini.^169,170^

Along with p53 antibodies, circulating CTL and CD4+ p53 specific cells have been detected in cancer patients.^171–173^ While mutp53 is known to promote an immunosuppressive role downregulating MHCI and upregulating immunosuppressive ligands such as PD-L1,^174^ a cell-mediated innate immune response is present in TNBC patients. TNBC patients with mutp53 had a higher immune score, including increased lymphocyte infiltration compared to patients with WT p53.^175^ It can be inferred that the increased accumulation of p53 will increase the presentation by MHCI and MHCII and thus recognition by these p53 T-cells. While mutp53 can lead to the activation of CTLs, this is not necessarily correlated with p53’s increased levels.^176^ Interestingly, the structural/destabilizing p53 mutations were more readily recognized by p53 specific T-cells compared to the contact mutations.^176^ Two major mechanisms of p53 presentation have been discovered. First, cancer cells can present p53 as an antigen for CD8+ T-cell recognition via MHCI. Second, mutated p53 can also be released by the necrotic regions of the tumor allowing it to be taken up and presented by APCs via MHC class I and II for CD4+ T-cell recognition.^161^

Due to the increased accumulation of p53 upon its mutation, p53 has been investigated as an antigen for immune therapy, with pre-clinical p53 vaccine studies beginning in the 1990s, predominantly with peptide-based vaccines.^177–180^ In 2002, the first clinical trial using a p53 vaccine was conducted utilizing a full length (FL) wild type (WT) p53 recombinant adenovirus vaccine.^181^ The vaccine was given to 6 late-stage cancer patients and an increase in humoral and anti-adenoviral immune responses were demonstrated, however no p53 specific antibodies or CTLs were detected.^181^ Since then, over 22 clinical trials with p53 vaccine in cancer have been conducted using recombinant viruses expressing WT p53, peptide tetramers or dendritic cell autologous transfers^182^ (Table 1). Another p53 study in 2003^184^ used FL WT p53 and, again, lead to no p53 specific immune response. The first breast cancer p53 vaccine clinical trial was in 2004 using a p53 WT peptide in a dendritic cell platform,^185^ which demonstrated a p53 specific immune response. This study progressed to phase II in 2007 but never progressed to phase III^190^ due to only 8 patients having a p53 specific immune response and 11 patients have progressive disease. In 2018 and 2019, two different Phase I/II trials in various tumors including breast examined the efficacy of WT FL p53 in adenovirus-p53 transduced dendritic cells and Modified Vaccinia Ankara virus platforms, respectively.^203,205^

**Table 1.** Compilation of cancer clinical trials involving p53 vaccination found on clinicaltrials.gov and/or in literature.

Author/Year	Phase	NCT #	Delivery	Antigen	Disease	Immune Outcome	Tumour Outcome
Khleif/1999	II	NCT00019084	Short peptide-pulsed DC +GM-CS	Mutated p53 Ras	Advanced Cancer	Paper focused on Ras.^183^	
Kuball/2002	Pilot		Recombinant Adenovirus	WT FL p53	Urogenital, lung cancer, malignant schwannoma	No p53-specific Abs or CD8+ detected, Abs, CD4+ and CD8+ for adenovirus detected^181^	No objective response observed
Menon/2003	I/II		Recombinant canarypox virus	WT FL p53	Colorectal cancer	No pathological auto-immune response^184^	Disease Progression
Svane/ 2004	I		Short peptide-pulsed DC	WT p53 peptide	**HLA-A2+ breast cancer**	T-cell response against p53 mutated and unmutated peptides^185^	Mixed response including disease stabilization and progressive disease
Lomas/2004	I		Short peptides plus GM-CSF	Short peptides derived from human anti-p53 (WT denatured) antibodies	**Breast**, colorectal, non-small-cell lung, renal, prostate, head and neck, hemangiopericytoma, esophageal cancer	No measurable IFNγ T-cell response against p53.^186^	Not measured and/or reported.
2000-2005	II	NCT00019929	Short peptide-pulsed DC	Mutant p53 peptides	**Stage III Non-Small Cell Lung** **Cancer**	NA	NA
Carbone/2005		NCTT93-0148	Peptides	Mutant p53 or K-ras	**Various Cancers**	Detection of cytotoxic T-cells in 4/9 patients in the adjuvant setting and 6/28 patients with evident disease IFNγ response was observed in 8/9 patients treated adjuvantly and in 8/ 28 patients treated with evident disease.^187^	No correlation of vaccination and improved clinical outcome.
Antonia/2006	I/II	NCT00049218	Recombinant adenovirus-transduced DC	WT FL p53	Small cell lung cancer	57.1% of patients had a significant p53 response.^188^	21/29 patients had progressive disease, 7 had stable and 1 achieved a partial response
Abstract: Herrin/ 2007 & Paper: Rahma 2012	II	NCT00019916	Short peptide-pulsed DC	WT p53 peptide 264–272	HLA-A2+ ovarian cancer	6/9 patients with subcutaneous injection and 5 of 6 with IV injections produced a p53:264-272 peptide specific immune response.^189^	No significant difference in PFS and OS between treatment groups. Only 1/20 patients were reported having no disease recurrence.
Svane/ 2007	II		Short peptide-pulsed DC	3 WT p53 peptides + 3 mutant p53 peptides	**HLA-A2+ breast cancer**	8/22 patients had p53 specific CTLs.^190^	Out of 19 observed patients, 8 had stable disease and 11 had progressive disease.
Leffers/ 2009	II		Synthetic Long Peptide (p53-SLP)	10 long peptides covering WT p53 (70-248)	Ovarian cancer	9/17 patients had p53 specific T-cells. All patients you received 4 immunisations had an IFNγ response to p53. Th2 dominant response.^191^	2 patients only received 2 vaccinations do to rapidly progressing disease. Of the patients who received 4 immunisations, 2/20 had stable disease and 18/20 had progressive disease.
Speetjens/2009	I/II		Synthetic Long Peptide (SLP)	10 long peptides covering WT p53 (70-248)	Colorectal cancer	Only CD4+ p53 specific T-cells were detected. Mixed Th1 and Th2 response.^192^	Not reported.
Yoo/ 2009	II		Recombinant Adenovirus	WT FL p53	Squamous cell carcinoma	Not reported.^193^	Cannot conclude due to size of study.
Trepiakas/ 2010	I/II	NCT00978913	Short peptide-pulsed DC	Short peptides for p53, Survivin, telomerase long peptide	Melanoma	6/10 patients had immune response to 5 peptides.^194^	No correlation between immune response induction and disease stabilization. 11/36 patients had stable disease.
Vermeji/ 2012	II	NCT00844506	Synthetic Long Peptide (SLP)	10 long peptides covering WT p53 (70-248)	Ovarian Cancer	90% of patients had an immune response to p53 vaccinations. P-53 specific T-cells were produced both Th1 and Th2 cytokines.^195^	2/10 patients had stable disease while 8/10 had progressive disease.
Ellebaek/2012	II	NCT00197912	Short peptide-pulsed DC	Survivin, hTERT, and p53-derived peptides Same treatment as previous study.^194^	Metastatic melanoma	2/7 patients had an immune response p53-derived peptides.^196^	Vaccine treatment did not induce any objective clinical responses.
Iclozan/ 2013	II		Recombinant adenovirus-transduced DC	WT FL p53	Small cell lung cancer	3/15 patients receiving peptide alone and 5/12 patients receiving peptide and ATRA had a IFNγ p53 specific response. Vaccine alone did not decrease MDSC levels nor increase granzyme-B+ CD8+ T cells.^197^	Vaccine alone has no immune response as shown previously.^188^ Further studies were performed to investigate the see efficacy of vaccine in increasing response to second-line chemotherapy.^198^
Zeestraten/ 2013	I/II		Long Peptide+IFN-α	10 long peptides covering WT p53 (70-248)	Colorectal cancer	Vaccine with IFN-α produced p53 specific T-cells.^199^	Not reported.
Hardwick/ 2014	I	NCT01191684	Modified vaccinia Ankara (MVA)	WT FL p53	Pancreatic cancer, colon cancer	Initial response to vaccine but no further expansion with further immunisations. T-cell and antibody responses to the MVA backbone.^200^	No detectable clinical responses.
Schuler/ 2014	I	NCT00404339	Short Peptide- pulsed DC	Short WT p53 peptides	HLA-A2+ HNSCC	11/16 patients had an increase in p53 specific T-cells with 4/16 having IFNγ secretion. Decrease in T-regs.^201^	Possible increase in DFS compared to previous studies.
Dijkgraaf /2015	I/II	NCT01639885	Long Peptide	10 long peptides covering WT p53 (70-248)	Ovarian Cancer	All vaccinated patients showed a p53 specific T-cell response.^202^	Some patients did not finish chemotherapy (gemcitabine) treatments. Partial response in 2 patients, stable disease in 4 patients and progressive disease in 10 patients.
2018/ Hardwick	I	NCT02275039	Modified vaccinia Ankara (MVA)	WT FL p53	Ovarian Cancer	Decrease in Tregs and MDSCs. 5/11 patients had an increase in p53 specific CD4+ and CD8+ T-cells.^203^	No complete responses. 3 patients had stable disease.
Soliman/ 2018	I/II		Recombinant adenovirus-transduced DC	WT FL p53	**Breast**, colon, gastric, lung, tongue, ovarian, chondrosarcoma cancer	7/23 patients had a >10% increase in CD8+ IFNy + cells.^204^	Stable disease in 4/39 patients. No difference in PFS between immunologic responders and non-responders.
Chiappori/ 2019	II	NCT00617409	Recombinant adenovirus-transduced DC	WT FL p53	Small cell lung cancer	Focused on clinical outcomes, as this was a continuation study from Iclozan 2013.^197^ 13/51 treated patients had an IFNγ immune response.^198^	Vaccination led to 1/45 patients having a partial response and 12/45 having stable disease. Study had many flaws and limitations leading to unconclusive results.
Chung/2019	I	NCT02432963	Modified vaccinia Ankara (MVA)	WT FL p53	**Breast**, pancreatic, hepatocellular, head and neck cancer	3 patients had a decreased CD4/CD8 ratio. 1 patient with TNBC who had pCR had a T-cell response correlating with clinical benefit.^205^	1 TNBC patient had a pCR, and another 6 TNBC patients were enrolled. 5 had disease progression and were removed from the study and 1 received all treatment and had stable disease for 30 weeks.
2018-2024 (Active)		NCT03113487	Modified vaccinia Ankara (MVA)	WT FL p53	**Ovarian, Primary Peritoneal, or Fallopian Tube** **Cancer**	NA	NA

FL = Full Length DC = Dendritic Cell pCR = Pathological Complete Response

Clinical trials including breast cancer patients are in bold.

These studies investigated the use of recombinant viral vectors, peptide pulsed dendritic cells, short peptide, and long peptide vaccines with limited success. In all, studies have shown that p53 vaccines can elicit an immune response (Table 1), however, do not improve patient survival enough to justify a phase III trial. We believe that p53 is still an antigen of interest for a breast cancer vaccine and a successful vaccine can be created provided further sequence and delivery optimization. To study the immune effect of p53 vaccines and impact of different antigenic regions, Bueter et al. designed 94 different peptides spanning the entire WT p53 protein and used these to stimulate PBMCs of colorectal cancer patients. They found that majority of p53 determinates elicited a Th2 response measured by IL-10, with the C-terminal region eliciting the most Th1 response measured by IFNγ.^161,206^

Therefore, one possible downfall to many of these clinical trials could be due to when the FL p53 is digested into peptides it promotes a greater Th2 vs Th1 response. We believe if optimization of the antigen sequence being delivered is performed, such as vaccination with only the C-terminal, this will allow for a more adaptive immune response, ideally activating cytotoxic T-cells for a Th1 dominant response. This is important in particular with p53, as anti-p53 antibodies, associated with a humoral response, have been associated with a poor prognosis in TNBC.^207^ Second, we believe by using an mRNA sequence of this peptide, there will be an increased immune response. mRNA vaccination has shown great success with the COVID-19 disease and p53 mRNA vaccines studies have been carried out.^208,209^ To date there has been 8 clinical trials using mRNA vaccines for breast cancer patients.^210^ Out of these studies, 2 incorporate mRNA p53. A completed study (NCT00978913) investigated a DC vaccine with p53 as one of its 3 antigens. The MERIT clinical trial (NCT02316457) discussed above also used p53 as one of their antigens.^210^ We are aware that further optimization is needed such as the carrier and administration route, however, with the developing mRNA and delivery system technology, we are optimistic about a future p53 vaccine for TNBC.

## Current limitations and future directions of TNBC vaccines

Due to the high heterogeneity of TNBC, it is difficult to find a universal antigen that can be effective in all patients. Also due to the aggressive nature of TNBC, past chemotherapy treatment has most likely been used changing the immune microenvironment, and further chemotherapy will be needed. Therefore, studies will be needed investigating the best chemotherapy to combine with vaccination to boost the immune system rather than deplete essential anti-cancer immune cells. As proposed in this review, p53 could be successful option for the treatment of TNBC. This again is firstly due to its high mutation rate in TNBC (up to 80%.^143,144^ Furthermore, while p53 vaccination has lacked success in breast cancer (Table 1), TNBC is more immunogenic than other subtypes,^39^ possibly increasing the chances of success. This increased immunogenic profile will allow the p53 vaccine to be paired with other immunotherapies, such as ICI, increasing success rates. As mentioned, studies will be needed to optimize combination with chemotherapy, similar to the use of gemcitabine in combination with p53 vaccination (NCT01639885, Table 1). Finally, the use of mRNA technology could lead to further success, as will now be discussed.

As seen throughout this review, there are various types of cancer vaccines including peptide-based, cell-based, nucleotide-based, and viral vector-based, each with its own strengths and limitations. The advantages and disadvantages of each type of vaccine has been previously well documented,^101^ therefore this section will focus on the benefits of mRNA vaccines and how they could lead to further vaccination success. Two advantages of mRNA vaccines over DNA vaccines are i) mRNA has no chance of incorporating into the genome and ii) mRNA only needs to enter into the cytoplasm and undergo translation in comparison to DNA vaccines needing to enter the nucleus for transcription. As mentioned in the above section, one advantage of mRNA vaccines over whole protein vaccines is the ability to choose which peptides will be displayed by APCs and skew the immune response toward Th1. Other advantages of mRNA are that the sequence can be modified to decrease the rate of degradation and thus increased translation. Modifications can also help avoid unwanted activation of the innate immune system, such as toll-like receptors 3,7 and 8, caused by the RNA.^211^ Possible pitfalls that come with mRNA vaccines is that during synthesis, in vitro transcription (IVT), mRNA is vulnerable to degradation which can lead to exogenous mRNA fragments, dsRNA and other contaminants which can cause unwanted immunogenicity. However, this can be mitigated through optimization of IVT protocols. For these reasons we believe mRNA is a great strategy for the development of a cancer vaccine.

There are various strategies for the delivery of mRNA, that again cannot be fully covered in this review, however literature has covered this extensively.^212^ A few delivery strategies include mRNA loaded lipid-based nanoparticles (LNPs), dendritic cells (DC) and cell-penetrating peptides (CPPs). Due to mRNA’s negative charge, the formation of nanoparticles using cationic lipids and peptides can be used to form nanoparticles. LNPs are able to interact easily with cell membranes and can protect the mRNA from degradation from endosomal enzymes.^213^ A great example of this is the successful mRNA COVID vaccines produced by Pfizer and Moderna.^214^ Similarly, the design of specific peptides, CPPs, that are able to escape the endosome and release the mRNA into the cytosol have been of great interest. One example of this is the RALA peptide that has been used for both mRNA delivery^215^ as well as drug delivery.^216^ An alternate method of delivering mRNA is through the pulsed of DC, which has been heavily used in p53 peptide vaccination (Table 1). This same approach can be used but with mRNA, which ensures MHC presentation and cytokine secretion for a robust immune response.^213^ All of these strategies have their advantages and disadvantages, and more optimization is needed, but the future of mRNA vaccine delivery is bright.

## Conclusion

Triple negative breast cancer is a deadly disease in need of new therapeutic strategies. Research has turned to the use of immunotherapy to aid in the treatment of TNBC. Great advancements in the development of a TNBC vaccine have been made; however, there is still a lot to be desired. We highlighted why p53 is an excellent antigen for a TNBC vaccine, showcasing the already rich clinical history and translatability. We believe with the use of mRNA and new delivery strategies discussed, a p53 vaccine has the potential to reach a larger patient population and translate to other cancers.
